# The Bright Side of Grit in Burnout-Prevention: Exploring Grit in the Context of Demands-Resources Model among Chinese High School Students

**DOI:** 10.1007/s10578-020-01031-3

**Published:** 2020-07-28

**Authors:** Ziwen Teuber, Fridtjof W. Nussbeck, Elke Wild

**Affiliations:** 1 Department of Psychology, Bielefeld University, Universitaetsstr. 25, 33615 Bielefeld, Germany; 2 Department of Psychology, University of Konstanz, Konstanz, Germany

**Keywords:** Personality, Resilience, Life satisfaction, Academic achievement, Chinese learners

## Abstract

This study contributes to understanding students’ emotional responses to academic stressors by integrating grit into the well-established Job Demands-Resources Model and by examining the relationship between academic demands, grit (consistency of interests, perseverance of effort), burnout, engagement, academic achievement, depression, and life satisfaction in Chinese students. We conducted a self-report study with *N* = 1527 Chinese high school students (*M*_age_ = 16.38 years, *SD *= 1.04). The results of structural equation modeling showed that after controlling for gender, socio-economic status, and school types, demands positively related to burnout and negatively related to engagement. Both facets of grit negatively related to exhaustion, whereas only perseverance of effort positively related to engagement. Burnout positively related to depression and negatively related to life satisfaction, whereas engagement positively related to life satisfaction. However, neither burnout nor engagement was related to academic achievement. Our findings indicate that grit may be protective against school burnout.

## Introduction

Shanghai’s outstanding performance at its first participation in PISA 2009 (Programme for International Assessment) triggered “a global ‘PISA-shock’ that repositioned this system as a significant new ‘reference society’, shifting the global gaze in education from Finland to the ‘East’ at the beginning of the so-called ‘Asian century’” [1, p. 464]. Thenceforth, Shanghai’s success has strongly influenced not only other national systems but also global education policies [1]. Although China’s education system seems to have immense potential, it also entails risks [2].

The main concern is the high level of academic demands in the Chinese education system. In China, access to higher education is determined by the yearly *gaokao* (National College Entrance Examination). *Gaokao* is the most important event for Chinese high school students and their parents and is considered as a test that determines their “destinies” because higher education is perceived as the only way to achieve socio-economic advancement. Although admission rates are increasing because of the general expansion of higher education, the gap in college admission in terms of social class and geography is still large [3]. According to the statistics provided by the Ministry of Education of China, the admission rate by Chinese universities in 2018 was only 40%. To maximize their odds to be selected by a(n elite) university, Chinese high school students (i.e., 10th–12th-graders) spend almost all their time on learning activities [4]. Additionally, due to the one-child policy in the last 35 years, school children face high educational expectations from their families (for further reading, see [5]). Thus, high school students in China experience enormous academic demands from many directions.

In school contexts, high academic demands are shown to be a significant risk factor for burnout [6], which rapidly increases the risk of dropping out of school [7] and the development of mental health problems [8]. According to the well-established *job demands-resources* (JD-R) [9] model, the availability of resources (i.e., protective factors) is crucial for coping with demands [9]. In contrast to Chinese students’ high academic performance in PISA studies, their well-being is rather low [10]. Compared to students from other OECD countries, Chinese students report more internalized symptoms. The high demands of the Chinese education system give ground for concern, and it is worth an in-depth examination of the relationship between high academic demands and emotional responses. Identifying protective factors that foster student academic and psychological adjustment may be valuable for researchers and practitioners to derive burnout prevention and intervention approaches.

In this study, we are interested in Chinese students’ *grit* which is defined as perseverance of effort and passion for long-term goals by Duckworth et al. [11]. We chose this construct because it is close to the learning value of Chinese students being strongly influenced by the Confucian tradition. Chinese learners view learning as a process of moral striving [12] and regard perseverance of effort in pursuing goals as the key to success [13, 14], which is also reflected in the old Chinese saying “zhi yao gong fu shen, tie chu mo cheng zhen” (“if you work at it hard enough, you can grind an iron rod into a needle” [15, p. 28]). In the last decade, grit has been found to positively affect one’s work/learning behavior and occupational/academic outcomes.

Using the JD-R model as a framework model, we explored the influence of grit on students’ emotional responses to academic demands, on their learning behavior, and consequently on their academic achievement and mental health.

## School Burnout

Burnout is generally defined as a work-related syndrome of emotional exhaustion, cynicism, and reduced personal accomplishment [16]. An increasing number of researchers have identified the analogous syndrome in students (for an overview, see [8]). In the school context, emotional exhaustion refers to a lack of energy or feelings of being emotionally overextended in the learning process. Cynicism means a detached attitude towards school (e.g., perceiving learning as senseless). Finally, reduced personal accomplishment describes one’s negative beliefs in academic competencies and achievement [17, 18].

However, concerning the three-dimensional structure of burnout, several authors (e.g., [19]) shared the opinion of Maslach et al. [16, p. 402] that emotional exhaustion was “the central quality of burnout” and “the most obvious manifestation” of this syndrome. Leiter and Maslach [20] proposed a model that indicated that emotional exhaustion was the first phase of burnout and led to cynicism (as a maladaptive coping strategy) as well as reduced personal accomplishment. Longitudinal studies provided evidence supporting this model in both job (e.g., [21]) and school contexts (e.g., [22]).

Like job burnout, school burnout can lead to serious mental and academic problems. Burnout is considered as an antecedent of depression (e.g., [23]) which has been ranked the fourth leading cause of illness and disability worldwide among adolescents aged 15–19 years by the World Health Organization (WHO). A recent report [24] on the prevalence rate of depression among Chinese adolescents shows worrying results (20% in 2012). In the school context, results of several longitudinal studies indicate that school burnout predicts depression (e.g., [25]). Besides depression, burnout has been linked to negative academic outcomes. In a longitudinal study, Bask and Salmela-Aro [7] revealed that burnout symptoms increased over time in high school and that cynicism, in particular, predicted school dropout. Compared to students with a low level of cynicism (i.e., the lowest 10th percentile), students with a high level of cynicism (i.e., the highest 10th percentile) were four times more likely to drop out of school.

In the occupational context, burnout is the result of prolonged and intensive exposure to stressors [16]. Likewise, education researchers found that exposure to high academic demands increases the likelihood of burnout syndrome in Finnish secondary school students [6], Chinese college students (e.g. [17]), and German university students [26].

## The JD-R Model in Job Contexts

In the past two decades, the JD-R model [27] has become a very popular heuristic model to predict job burnout and has been used to derive prevention and intervention approaches. Strong cross-sectional and longitudinal evidence supports its applicability and flexibility across a wide variety of occupational settings (for an overview, see [28]).

Based on the assumption that specific physical, psychological, emotional, and organizational aspects of the job have positive or negative effects on occupational and psychological outcomes, the JD-R model divides job characteristics into job demands and job resources. Job demands require physical and psychological effort, while job resources refer to those that “(a) [are] functional in achieving work goals; (b) reduce job demands and the associated physiological and psychological costs; or (c) stimulate personal growth, learning, and development” [9, p. 9]. The two categories of job characteristics evoke two independent psychological processes. In the so-called *health impairment* process, job demands determine the strain and, later, diminish mental health. In the *motivational* process, job resources lead to more job engagement and, later, facilitate job performance. In addition to the main effects of job demands and job resources, the JD-R model posits two interaction effects. On the one hand, job resources buffer the detrimental effect of job demands on stress reactions. On the other hand, the positive effect of resources on engagement can be pronounced if demands are high.

The initial JD-R model has been restricted to occupational characteristics and neglected personal resources, which can play an important role during employees’ adaption to job demands [29]. Thus, some researchers have extended this model by including personal resources [30]. The role of personal resources in the JD-R model is, however, empirically still unclear. Their moderating effects are especially inconsistent. Xanthopoulou et al. [30] found that personal resources (self-efficacy, organizational-based self-esteem, and optimism) affected the perception of job resources and mediated the relationship between job resources and exhaustion/engagement, whereas personal resources did not moderate the relationship between perceived job demands and exhaustion. On the contrary, some studies (e.g., [31]) found, besides the main effects, the interaction between job demands and personal resources on emotional exhaustion and engagement.

## The JD-R Model in School Contexts

Salmela‐Aro and Upadyaya [6] used the JD-R model to predict school burnout and schoolwork engagement. Schoolwork engagement was defined by the authors as a motivational state of *energy* (characterized by high levels of vigor and mental resilience while learning), *dedication* (a positive cognitive attitude towards learning, interest in one’s academic work), and *absorption* (feelings of competence and success) in schoolwork. About 1700 Finnish adolescent students (*M*_age_ = 15.5 years) participated in this longitudinal self-report study. The results showed that perceived academic demands significantly predicted school burnout, whereas perceived personal resources predicted higher levels of engagement and lower levels of burnout. Burnout and engagement positively predicted depressive symptoms and negatively predicted life satisfaction. Finally, school burnout mediated the relationship between academic demands and mental health outcomes.

The JD-R model was also applied to German secondary school students [32]. The results support the assumed dual processes: Academic demands (e.g., academic overload) positively related to burnout and negatively related to schoolwork engagement, whereas students’ resources (e.g., optimism and integration into peers) positively related to engagement and negatively related to burnout. Moreover, students who reported high levels of burnout tended to show poorer academic performance. Yet, there were no significant interactions between demands and resources.

Hence, both studies provide evidence for the transferability of the JD-R model to school-related contexts. However, further empirical studies are necessary to understand the impact of academic demands and students’ resources on their emotional responses and engagement, and finally, on their mental health and academic achievement.

## Grit as a Predictor of Achievement

In the Western research community, non-cognitive predictors of high achievement have been of great interest for several decades. For instance, 40 years ago, Renzulli [33] emphasized the important role of task commitment and creativity besides ability. More recently, American researchers introduced the trait-like construct *grit* and defined it as “perseverance and passion for long-term goals” [11, p. 1087]. According to Duckworth [11], grit consists of two dimensions: *consistency of interests* and *perseverance of effort*. Consistency of interests refers to the tendency to change interests and goals seldomly. Perseverance of effort refers to the tendency to work hard even in difficult situations. According to the authors, gritty individuals show an enduring commitment to their ambitions, work strenuously towards challenges, and sustain interest and effort for a long time despite adversity. Serval studies provide evidence for the power of grit in predicting retention in the military, workplace, school, and even marriage [34–36].

Grit is predominantly operationalized as a higher-order construct (i.e., the overall grit scale [37]). Several scholars criticized this approach. A recent meta-analysis by Credé and colleagues [37] showed that using the overall grit score might impair the predictive power of this concept with respect to performance. Furthermore, the results of a large cross-cultural study by Disabato et al. [38] suggested that the use of the grit overall score was more appropriate in individualistic than in collectivistic cultures. Therefore, in the present study, grit is operationalized as two correlated first-order factors instead of one higher-order factor.

Apart from discussions on the measurement of grit, Credé et al. [37] pointed out that grit overlapped with other established constructs such as persistence, proactivity, industriousness, need for achievement, and (facets of) conscientiousness such as self-control or order. In particular, the relationship between grit and conscientiousness has been intensely debated. Schmidt et al. [39] analyzed the connection between grit and conscientiousness while accounting for the complex structure of conscientiousness and the two-dimensional conception of grit. The results of their study suggested that grit could be completely integrated into the hierarchical structure of conscientiousness. Similarly, Rimfeld et al. [40] found in their twin study that the etiology of grit and conscientiousness was highly similar. Opposing, Duckworth et al. [11, p. 1089] have argued that “grit overlaps with achievement aspects of conscientiousness but differs in its emphasis on long-term stamina rather than short-term intensity.” This argument was supported by Abuhassàn and Bates [41]: Conscientiousness was positively related to school grades, whereas perseverant grit was associated with higher life-course accomplishment. Moreover, Duckworth et al. [11] found in their study series that grit predicted success beyond IQ and conscientiousness. The incremental validity of grit for predicting academic performance beyond Big-Five personality traits (i.e., extraversion, agreeableness, conscientiousness, openness, and neuroticism), self-control, and intelligence was also supported in a Chinese sample [42].

In addition to these conceptual debates, it has been questioned if grit is always beneficial. In daily life, it is argued that, sometimes, it may be better to adapt one’s strivings to situational demands or circumstances. Perseverance may also be detrimental if individuals strive for goals that challenge or even exceed their individual or environmental resources. In the school context, Shechtman et al. [43, p. 42] point out that, “promoting perseverance for goals inappropriate for the students can induce stress and have detrimental long-term effects.” However, as stated above, grit and also its two constituents—effort and high academic goals—are highly valued in the Chinese culture. The present study, therefore, focuses on the assumed positive function of grit for students’ academic and psychological adjustment.

### Integrating Grit into the JD-R Model

**Fig. 1:**
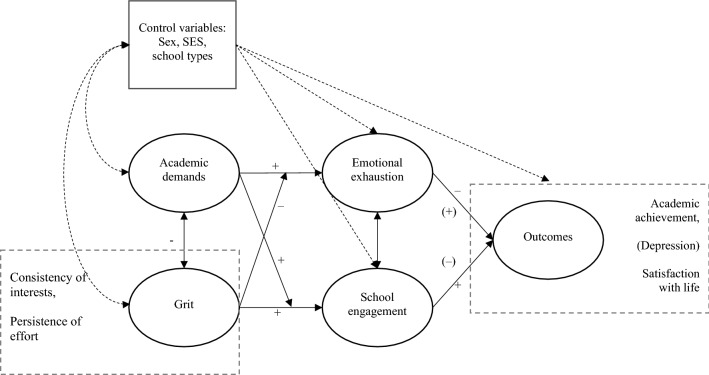
Locating the variables of interest in the JD-R model. Assumed effects of exhaustion and engagement on depression are presented in parentheses. Dotted paths = not included in the original JD-R. *SES* socio-economic status

In the current study, we use the JD-R model as a guiding framework to explore the role of grit (Fig. 1). From this perspective, demands are seen as the perceived difficulty and quantity of academic tasks, while grit is seen as a personal resource that is functional in dealing with these tasks. The operationalization of schoolwork engagement (energy, dedication, and absorption) is based on Salmela-Aro and Upadaya [44].

#### The Dual Processes

Gritty people pursue their long-term goals with passion; passion for long-term goals fuels achievement because gritty people tend to utilize self-control to sustain their focus on goals in the face of challenging or stressful conditions [45]. Bowman et al. [46] revealed that gritty American college students engaged more in college activities and were less likely to change majors than their less gritty peers. Further, Datu et al. found that grit (overall scale) positively predicted positive emotions and well-being among Filipino high school students [47] and Chinese primary school students [48]. Hence, it is reasonable to assume that gritty students tend to engage more in schoolwork and feel less emotionally exhausted. We expect a high level of academic demands to be related to a higher level of school burnout, whereas burnout and schoolwork engagement are expected to be negatively associated.

#### The Presumed Mediating Role of Emotional Exhaustion and Engagement

The JD-R model suggests that burnout detrimentally affects one’s achievement and mental health (e.g., depression) [49]. This assumption has been supported not only by Western researchers [25] but also by Asian researchers [50, 51]. Based on this theoretical reasoning, we envisage that school burnout mediates the relationship between academic demands and depressive symptoms.

According to the JD-R model, engagement predicts well-being outcomes (e.g., life satisfaction; [52]) and higher academic achievement. In line with the JD-R assumptions, Bowman et al. [46] found that American college students’ higher commitment and engagement predicted their satisfaction and greater academic achievement. An Australian study [53] also highlights the mediating role of engagement between grit and academic achievement. This finding could be replicated in Filipino [54] and Chinese school students [55]. Thus, we expect that gritty students engage more in schoolwork, and in turn, show higher academic performance and are more satisfied with their life.

#### Interactions Between Demands and Grit

Another assumption of the JD-R model is that there are interaction effects of job demands and job resources on emotional exhaustion and work engagement. We assume that grit has a stronger impact on school engagement when perceived academic demands are high. Gritty students cope better with their academic demands, and thus grit buffers the negative effect of high academic demands.

### Hypotheses

Based on the framework delineated above, we formulate the following hypotheses:

- Ha1: High academic demands are positively related to emotional exhaustion and negatively related to schoolwork engagement, whereas grit is negatively related to exhaustion and positively related to schoolwork engagement.
- Ha2: Schoolwork engagement is positively related to academic achievement and life satisfaction and negatively related to depression, whereas exhaustion is positively related to depression and negatively related to satisfaction with life.
- Hb-extended Ha: The positive relationship between academic demands and emotional exhaustion becomes weaker if the level of grit is high. The positive relationship between grit and schoolwork engagement becomes stronger if the level of academic demands is high.
- Hc: Exhaustion and schoolwork engagement mediate the relationships between demands/grit and psychological as well as academic outcomes: Higher levels of demands are related to higher levels of exhaustion, which are in turn related to higher levels of depression and lower levels of academic achievement and life satisfaction. Higher levels of grit are related to higher levels of engagement, which are in turn related to higher levels of achievement and life satisfaction as well as lower levels of depression. We will *explore* this theoretically founded hypothesis based on cross-sectional data admitting that mediation hypotheses cannot be *tested* relying on non-experimental cross-sectional data and that the results cannot be causally interpreted.

## Methods

### Data Collection

We focus on high school students (i.e., 10th–12th-graders) in Shanghai because students in Shanghai are confronted with double pressure: heavy competition within Shanghai (due to the high academic level in general) and competition from outside of Shanghai (the enrollment of a university in a big city as a chance of social advancement for rural students).

High schools in China are categorized into “key” and “ordinary” schools. In comparison to ordinary schools, key schools have a better academic reputation and are usually allocated more resources [56]. In Shanghai, 33% of high schools are Shanghai key high schools (most resources and highest academic demands), 40% district key high schools, and around 27% ordinary high schools (lowest resources and lowest demands). Against this background, we randomly involved one private ordinary high school, one ordinary high school, one campus district high school (where students have the opportunity to live on campus), one district key high school, and one Shanghai key high school to represent the differences in the Chinese school system.

Previous to the data collection (in February and March 2019), school principals were personally contacted and informed about the purpose of this study. The schools distributed a letter to the students and their parents or guardians explaining the nature and design of the study. All participants were informed that they were free to withdraw from participation at any time without consequences. The students responded anonymously and voluntarily to the survey. The present study has the approval of the ethics review committee of Bielefeld University.

### Participants

Participants were *N* = 1527 high school students (52.40% females; age span: 15–19 years) from 42 classes. Their mean age was 16.38 years (*SD* = 1.04). The participation rate was 80.79%. Around 11% of participants were from the private ordinary high school, ca. 44% from the ordinary high school, ca. 16% from the campus district key high school, ca. 9% from the district key high school, and ca. 20% from the Shanghai key high school. The socio-economic status of them was normally distributed. Thus, our participants could be regarded as representative of high school students in Shanghai. All participants were Mandarin native speakers.

### Measures

#### Academic Demands

Perceived academic demands were investigated with a Chinese four-item scale by Teuber et al. [4]. Two items were used to investigate the workload (“How do you evaluate the amount of homework?” and “How do you evaluate the frequency of exams?” 1 = *little*, 4 = *very much*). The remaining two items were used to measure the difficulty of learning materials (“How difficult is the schoolwork for you usually?” and “How difficult are the exams for you usually?” 1 = *very easy*, 4 = *very difficult*). In this study, a confirmatory factor analysis (CFA) supported the one-factor structure (χ^2 ^= 5.51, *df* = 1, *p* < 0.05, CFI = 0.99, SRMR = 0.01, RMSEA = 0.05, 90% CI [0.02, 0.10]). The internal consistency was high (McDonald’s omega = 0.80).

#### Grit

To assess grit, we used the Chinese version of the Short Grit Scale (Grit-S [57])**.** It comprised eight 5-point items (1 = *not at all like me*, 5 = *very much like me*). The validity of the Chinese version has been proven by Li and colleagues [42]. Four reverse-worded items described one’s consistency of interests (e.g., “I often set a goal but later choose to pursue a different one”) and four items described the tendency towards sustained effort (e.g., “I finish whatever I begin”). A CFA indicated that a model with two first-order latent factors (consistency and effort) fitted the data well (χ^2 ^= 50.41, *df* = 19, *p* < 0.001, CFI = 0.98, SRMR = 0.03, RMSEA = 0.03, 90% CI [0.02, 0.04]). McDonald’s omega of the two subscales was 0.75 and 0.76, respectively.

#### Emotional Exhaustion

Emotional exhaustion was measured with the emotional exhaustion subscale of the Chinese version [58] of the Maslach Burnout Inventory-Student Survey [59]. It consisted of three 5-point items (e.g., “I feel emotionally drained by learning”). Its psychometric properties have been confirmed in previous studies [4]. In the present analyses, a one-factor structure fitted the data well (χ^2 ^= 1.92, *df* = 1, *p* = 0.17, CFI = 1.00, SRMR = 0.03, RMSEA = 0.02, 90% CI [0.00, 0.08]). McDonald’s omega was 0.86.

#### Schoolwork Engagement

In line with previous research [44], we adapted the Chinese version of the Utrecht Work Engagement Scale (UWE; [60]) measuring work engagement by replacing “work/job” by “schoolwork” to assess schoolwork engagement. The concept of schoolwork engagement consisted of three dimensions with three items each: absorption (e.g., “Time flies when I’m studying”), vigor (e.g., “When I’m studying, I feel mentally strong”), and dedication (e.g., “I find my studies to be full of meaning and purpose”). Students responded on a 7-point scale (0 = *never*; 6 = *always*). Although the scale was developed to differentiate between these three dimensions, the original authors recommend a one-factor model [44]. Likewise, the result of model comparison (Δχ^2^ = 208.95, *df* = 2, *p* < 0.001) indicated that the one-factor model (χ^2 ^= 188.95, *df* = 26, *p* < 0.001, CFI = 0.97, SRMR = 0.03, RMSEA = 0.06, 90% CI [0.06, 0.07]) fitted the data better than the three-factor model (χ^2 ^= 379.90, *df* = 24, *p* < 0.001, CFI = 0.92, SRMR = 0.05, RMSEA = 0.10, 90% CI [0.09, 0.11]). Therefore, we used the overall score (McDonald’s omega = 0.94).

#### Depressive Symptoms

Depressive symptoms were examined with the Chinese version of the Center for Epidemiologic Studies Depression Scale (CES-D [61]). The scale was designed for use in studies on the general (non-clinical) population. Previous analyses support a four-factor structure (Depressed Affect, Positive Affect, Somatic Symptoms/Retarded Activity, and Interpersonal) that can be represented by 20 4-point items (e.g., “During the past week, I felt sad.” 0 = *rarely or none of the time*, 3 = *most or almost all the time*). The sum score ranged from 0 to 60. A score equal to or above 16 indicated a person at risk for clinical depression. In Chinese adolescents, the Chinese CES-D shows very good psychometric properties [62]. The 4-factor structure could be confirmed in our study (χ^2 ^= 1086.75, *df* = 166, *p* < 0.001, CFI = 0.91, SRMR = 0.05, RMSEA = 0.06, 90% CI [0.06, 0.06]). In this study, we used the sum score (McDonald’s omega = 0.94).

#### Life Satisfaction

Life satisfaction was assessed using the validated Chinese version [63] of the Satisfaction with Life Scale (SWLS; [52]). The SWLS consisted of five items (e.g., “In most ways, my life is close to my ideal.” 1 = *strongly disagree*, 7 = *strongly agree*). In this study, this scale showed a unidimensional factor structure (χ^2 ^= 56.30, *df* = 5, *p* < 0.001, CFI = 0.99, SRMR = 0.02, RMSEA = 0.08, 90% CI [0.07, 0.10]). McDonald’s omega was 0.93.

#### Academic Achievement

Academic achievement was measured by two items. Students were asked about their sum scores of three main courses (i.e., Chinese, Mathematics, and English) in the last term exams. To compare the scores, we standardized the exam scores in percentages; for instance, 90% means that a student has achieved 90% of the full score.

#### Demographics/Control Variables

Demographic variables included: sex (0 = *male*, 1 = *female*) and school types (two dummy-coded variables: 0 = *not district key high schools*, 1 = *district key high schools*; 0 = *not Shanghai key high schools*, 1 = *Shanghai key high school*). To assess socio-economic status (SES), we asked for the number of books in the home using the same item as in the Chinese PISA study [64] (1 = *less than 20 books,* 5 = *more than 200 books*). A huge number of studies [65] shows that this item is strongly correlated with parents’ income and educational level and can, thus, be seen as a powerful indicator of SES.

### Analytic Strategy

Descriptive analyses included means, standard deviations, and inter-correlations. The JD-R has not been tested in Chinese student populations. Hence, we did not have population parameters on the presumed effects, the communality across the measured variables, and the degree of factor determinacy that would be required for a sound a priori power analysis [66]. According to various rules-of-thumb (for an overview, see [67]), our sample size can be regarded as large which should allow for a sound estimation of all model parameters.

To test the hypotheses, we specified structural equation models (SEM) in *Mplus 8* [68]. We used the TYPE = COMPLEX (CLUSTER = class) option to respect the clustered data structure. Missing value analysis indicated that, for all variables, data were missing between 0.3 and 22% of the cases. Little’s test (LIT) did not reach significance (χ^2 ^= 253.57, *df* = 230, *p* = 0.14) pointing to a Missing Completely at Random (MCAR) mechanism and allowing us to use the MLR as full-information estimator, which combines parameter estimation with missing data analysis and is robust to nonnormality of observed variables.

In order to create homogeneous metric indicators (parcels) for the latent variables, we applied the commonly used item-to-construct balance technique according to factor loadings [69] with the exception of emotional exhaustion, academic achievement, and depression. Because there were only three items measuring emotional exhaustion, we used these three items as indicators. We incorporated the control variables, depression, and academic achievement as manifest variables into the SEM.

To test our hypotheses Ha and Hb, we followed a stepwise analysis plan: We first tested the more general Hb by estimating an SEM with latent interactions using the LMS-approach [70] based XWITH option in *Mplus* (demands × consistency of interests; demands × perseverance of effort). In the second step, we ran an SEM including all main effects (significant and non-significant) and (only) the significant interaction effects of step 1 resulting in the final model including all presumed main effects (Ha) and the significant interaction effects (Hb). To test Hc, we added multiple mediators to the SEM. Bootstrapping procedures in *Mplus* were used to test the significance of the mediation effects. In this study, 5000 bootstrapping samples were generated from the original data set by random sampling. In all analyses, we controlled for gender, SES, and school types (i.e., modeled as covariates).

To evaluate the model fit, we relied on the recommendations by Hu and Bentler [71] with a non-significant χ^2 ^–value, a Comparative Fit Index (CFI) ≥ 0.95, Root Means Square Error of Approximation (RMSEA) ≤ 0.05, and Standardized Root Mean Square Residual (SRMR) ≤ 0.05 indicating good model fit and CFI ≥ 0.90, RMSEA ≤ 0.08, and SRMR ≤ 0.08 indicating acceptable fit. For model comparisons, we used Akaike information criterion (AIC) and Bayesian information criterion (BIC): the lower AIC and BIC, the higher the quality of the model [72].

## Results

Table 1 reports descriptive statistics for the total sample and for the five high schools. Table 2 shows Pearson’s correlations of all study (manifest) variables.

**Table 1:** Means and standard deviations (in parentheses) for the total sample and the five high schools, respectively

	DE	INT	PER	EE	ENG	DEP	SWLS
Total sample	2.64 (0.49)	2.95 (0.79)	3.10 (0.76)	3.01 (1.11)	3.06 (1.19)	17.64 (11.20)	4.31 (1.43)
Private ordinary high school	2.61 (0.53)	2.86 (0.90)	3.18 (0.83)	3.04 (1.17)	3.05 (1.34)	19.05 (11.68)	4.36 (1.43)
Ordinary high school	2.56 (0.47)	2.94 (0.81)	3.11 (0.79)	3.00 (1.10)	3.04 (1.22)	18.17 (11.38)	4.27 (1.45)
Campus district key high school	2.73 (0.45)	2.88 (0.77)	3.03 (0.67)	3.34 (1.04)	3.00 (1.14)	17.72 (11.06)	4.02 (1.49)
District key high school	2.52 (0.48)	3.01 (0.70)	3.01 (0.71)	2.92 (1.00)	2.94 (1.01)	20.11 (10.9)	4.42 (1.35)
Shanghai key high school	2.81 (0.47)	3.06 (0.74)	3.15 (0.73)	2.82 (1.13)	3.22 (1.15)	14.58 (10.13)	4.54 (1.34)

*DE* perceived academic demands, *INT* consistency of interests, *PER* perseverance of effort, *EE* emotional exhaustion, *ENG* schoolwork engagement, *DEP* depressive symptoms, *SWLS* satisfaction with life

**Table 2:** Correlations between all scale scores

	DE	INT	PER	EE	ENG	ACH	DEP
INT	−0.05*						
PER	− 0.02	0.14***					
EE	0.32***	− 0.21***	− 0.23***				
ENG	− 0.14***	0.13***	0.56***	− 0.29***			
ACH	0.03	0.07*	0.09**	− 0.05	0.13***		
DEP	0.09***	− 0.26***	− 0.34***	0.40***	− 0.28***	− 0.34***	
SWLS	− 0.05	0.11***	0.40***	− 0.26***	0.38***	0.40***	− 0.49***

*DE* academic demands, *INT* consistency of interests, *PER* perseverance of effort, *EE* emotional exhaustion, *ENG* schoolwork engagement, *ACH* academic achievement, *DEP* depressive symptoms, *SWLS* satisfaction with life. *p < 0.05. **p < 0.01. ***p < 0.001

### Results Regarding Ha and Hb

**Fig. 2:**
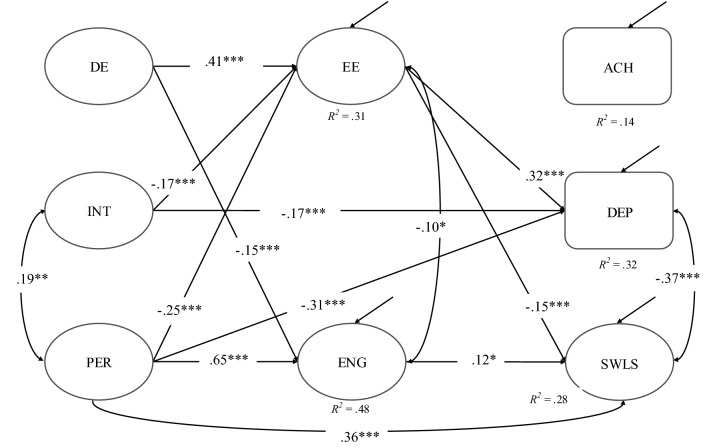
Final SEM with significant standardized regression coefficients after controlling for sex, school type, and SES. *DE* academic demands, *INT* consistency of interests, *PER* perseverance of effort, *ENG* schoolwork engagement, *EE* emotional exhaustion, *ACH* academic achievement, *DEP* depressive symptoms, *SWLS* satisfaction with life. *p < 0.05. **p < 0.01. ***p < 0.001. Note: For sake of simplicity, control variables and their path coefficients as well as non-significant path coefficients are not depicted but estimated in the model

Following the data analysis plan, we first specified SEM including latent interactions of demands with grit subscales. It turned out that none of the latent interaction terms reached significance: perseverant grit × demands on exhaustion (β = -0.03, *p* = 0.41) and on engagement (β = 0.02, *p* = 0.37); consistency of interests × demands on exhaustion (β = 0.00, *p* = 0.92) and engagement (β = 0.02, *p* = 0.74). Model comparisons between the model including interaction terms (AIC = 70,861.48, BIC = 71,393.80) and the model without any interaction (χ^2 ^= 330.56, *df* = 104, *p* < 0.001, *CFI* = 0.97, *SRMR* = 0.03, *RMSEA* = 0.04, 90% *C.I.* = [0.03,0.04], AIC = 70,855.45, BIC = 71,366.48) indicated that the latter model fitted the data equally well. Hence, we interpret the latter model as depicted in Fig. 2.

Overall, 32% of the variance in emotional exhaustion, 48.2% of the variance in schoolwork engagement, and 27.9% of the variance in life satisfaction could be explained. Exhaustion was positively related to academic demands (β = 0.41, *p* < 0.001). Respondents who reported high levels of consistency of interests (β = -0.17, *p* < 0.001) and high levels of the perseverance of effort (β = − 0.25, *p* < 0.001) reported lower levels of exhaustion. The relationship between engagement and perseverant grit was positive (β = 0.65, *p* < 0.001), whereas no relationship between engagement and consistency of interests was found (β = 0.04, *p* = 0.11). Further, students who reported high levels of engagement also reported higher levels of life satisfaction (β = 0.12, *p* < 0.05), however, its relations to academic achievement (β = 0.08, *p* = 0.14) and depressive symptoms were not significant (β = 0.03, *p* = 0.48). Exhaustion and depressive symptoms were positively related (β = 0.32, *p* < 0.001), no significant association was found between exhaustion and academic achievement (β = 0.01, *p* = 0.82).

### Results Regarding Hc

According to the results (Table 3), seven of the presumed 18 mediated paths were statistically significant: Engagement mediated the relationship between perseverant grit and life satisfaction (1); exhaustion mediated the relationships between demands and life satisfaction (2), between demands and depression (3), between perseverant grit and life satisfaction (4), between perseverant grit and depression (5), between consistency of interests and life satisfaction (6), between consistency of interests and depression (7).

**Table 3:** Standardized indirect effects (potential mediations) (B), standard error (SE), and 95% bootstrap confidence interval

Indirect effects	B	SE	95% CI	*p*
DE → EE → ACH	0.00	0.02	[− 0.03, 0.04]	0.92
DE → ENG → ACH	− 0.01	0.01	[− 0.03, 0.01]	0.17
DE → EE → SWLS	− 0.06	0.02	[− 0.10, − 0.03]	< 0.001
DE → ENG → SWLS	− 0.02	0.01	[− 0.04, 0.00]	0.07
DE → EE → DEP	0.13	0.02	[0.10, 0.16]	< 0.001
DE → ENG → DEP	− 0.01	0.01	[− 0.02, 0.01]	0.49
GPER → EE → ACH	0.00	0.01	[− 0.02, 0.02]	0.82
GPER → ENG → ACH	0.05	0.04	[− 0.03, 0.12]	0.15
GPER → EE → SWLS	0.04	0.01	[0.02, 0.06]	< 0.01
GPER → ENG → SWLS	0.08	0.03	[0.01, 0.14]	< 0.05
GPER → EE → DEP	− 0.08	0.01	[− 0.11, − 0.05]	< 0.001
GPER → ENG → DEP	0.02	0.03	[− 0.04, 0.08]	0.49
INT → EE → ACH	0.00	0.01	[− 0.02, 0.01]	0.82
INT → ENG → ACH	0.00	0.00	[0.00, 0.01]	0.32
INT → EE → SWLS	0.03	0.01	[0.01, 0.04]	< 0.01
INT → ENG → SWLS	0.00	0.00	[− 0.01, 0.01]	0.26
INT → EE → DEP	− 0.05	0.01	[− 0.07, − 0.03]	< 0.001
INT → ENG → DEP	0.00	0.00	[0.00, 0.01]	0.59

*DE* academic demands, *INT* consistency of interests, *PER* perseverance of effort, *EE* emotional exhaustion, *ENG* schoolwork engagement, *ACH* academic achievement, *DEP* depressive symptoms, *SWLS* satisfaction with life

## Discussions

In the current study, we consider grit as a personal resource and integrate it into the well-established JD-R model. Based on a Chinese high school student sample, we tested if academic demands were positively related to burnout and negatively related to academic achievement and mental health and if grit was positively associated with engagement, achievement, and mental health (main effects). Furthermore, we investigated if grit could buffer the detrimental effect of demands and boost engagement (i.e., interaction effects). Finally, we explored if burnout and engagement could be a mediator in the JD-R model. All of the analyses were controlled for sex, SES, and school types.

Most results are in line with our assumptions and previous findings. Perceived academic demands were positively related to emotional exhaustion and negatively related to schoolwork engagement. Both facets of grit (consistency of interests, perseverance of effort) were negatively related to emotional exhaustion. Further, perseverance of effort was positively associated with schoolwork engagement. We also found that exhaustion was positively related to depression and negatively related to satisfaction with life, whereas schoolwork engagement positively linked to life satisfaction.

The most unexpected result is that after controlling for sex, SES, and school types, academic achievement was no longer related to all other variables. This may be due to the way we assessed achievement: First, participants were asked to report on their exam scores and may intentionally over-inflate them. Second, the retrospective report may also lead to inaccuracy.

Regarding grit, both facets showed expected relationships with emotional exhaustion. However, we did not find any significant relationship between consistency of interests and schoolwork engagement. This result is consistent with prior studies (for an overview, see [37]) that show that consistency of interests has weaker effects on engagement than the other dimension of grit. From a cross-cultural perspective, we speculate that the role of consistency of interests for academic adjustment of Chinese students may be minimized because of the social and cultural consensus about the high value of effort and achievement in China. To achieve their academic goals, Chinese students may be willing to suppress their interests. However, this dimension may be relevant in individualistic cultures in which personal needs and interests are more important. In particular, various Western authors show that experiencing motivational conflicts (i.e., “want” vs. “should” conflicts) can interfere with one’s well-being and engagement (e.g., [73]).

As expected, gritty students experience less exhaustion and engage more in schoolwork. However, higher school demands are associated with higher levels of emotional exhaustion irrespective of the degree to which a student is gritty. One explanation for this observation is that gritty students are more likely to persist in demanding learning tasks [11] but are not necessarily the most intelligent ones. Further, due to the proactive nature, gritty students are more likely to receive positive responses from their teachers because they might be viewed as hard-working and self-disciplined. Thus, gritty students may have more environmental resources that influence the coping process. This could also explain why we did not find interaction effects between demands and grit. Grit may interact with other factors such as coping strategies. Gritty students who seek social support may cope with demands more successfully than those who choose avoidant coping strategies.

In our study, the path coefficient between engagement and depressive symptoms was not statistically significant, whereas the path coefficient between burnout and depressive symptoms was significantly positive. This supports recent findings in both work and school contexts [6, 74]: In the motivational process, personal resources increase engagement, which in turn is associated with positive outcomes (e.g., life satisfaction). In the mental health impairment process, demands increase the likelihood of strain, which in turn is associated with negative outcomes (e.g., depression).

The presumed mediating role of emotional exhaustion and engagement could not be strictly tested due to the cross-sectional data. Yet, we interpret our results as not contradicting the assumption that students who perceive high demands also experience more burnout, which in turn is related to more depressive symptoms and less life satisfaction. In this respect, perseverance of effort and consistency of interests may protect against being burned-out [25] and positively affect mental health [50, 51]. Perseverant students engage more in schoolwork, which in turn is positively related to life satisfaction. We assume that perseverant students are more motivated and believe more in their ability to control their lives than their peers. Thus, they are more likely to be satisfied. Yet, the presumed mediated effects with exhaustion and engagement as mediators could not be strictly tested in this study but have to be investigated relying on longitudinal data.

### Theoretical and Practical Significance

The current study offers further support for the applicability of the JD-R model in the Chinese school context. The JD-R may be used as a framework to understand Chinese students’ emotional responses to academic demands and to reveal resources that may prevent students from burnout.

With respect to grit, our findings suggest that both facets may be protective against school burnout. However, only perseverant grit (rather than consistency of interests) contributes to students’ involvement. The pattern of our findings advocates the criticism [40] that using a single global indicator (overall scale) of grit might disguise the effects of the distinct facets.

Corresponding results from our study and studies in different countries suggest that the relationships postulated in the JD-R model may be universal. However, this hypothesis needs to be tested in cross-cultural studies. Since most assumptions derived from the JD-R model were supported, this framework can be used to develop and implement interventions that reduce school burnout in China.

In line with previous findings, gritty students are less likely to be burned-out and more likely to engage in school-related activities. Successful adjustment facilitates their mental health and well-being. Therefore, the assessment of grit may be helpful to identify students at greater risk of burnout and motivational withdrawal. Less gritty students may profit the most from programs like imparting learning and coping strategies, time management, and smart goals. Furthermore, perseverance of effort may be promoted by parental involvement (more precisely: academic socialization [75]), such as communicating about the value of education and fostering educational aspirations (like most Chinese parents do).

Consistent with the model, the results show that high academic demands may be a risk factor for burnout. As early as the 1990s, China’s Ministry of Education was aware of the potentially detrimental effect of academic demands and proposed the *jianfu* rule (alleviating academic burden in basic education; e.g., reducing workload and test difficulty and downgrading the importance of scores) for the first time as a countermeasure. Yet, the educational pressure of families may impede the effect of this strategy: Many parents feel highly responsible for providing their children with more extracurricular learning to help them stand out from their peers and eventually be enrolled by an elite university. This is especially true for Chinese families. Yet, since the first launch of PISA in 2000, adolescents and parents in many PISA participating countries have also reported higher stress due to the increasing competition in education systems [10, 76, 77]. Against this background, our findings strongly encourage therapists and school psychologists to address the negative side of high academic demands and provide adolescents with accessible coping training or even integrate training into the curriculum (e.g., school physical education can be supplemented with relaxation techniques or mindfulness-based concepts).

### Limitations and Directions for Future Studies

Some limitations of this study must be noted. The major one is its cross-sectional design. Although the direction of the relationships is theoretically founded and supported by previous studies (e.g., [6]), future research should longitudinally investigate all presumed relationships especially with respect to the mediation hypotheses. Second, this study relied on students’ self-reports; common method variance may partly explain some of the results. It could be an advantage to obtain multiple measures of some constructs (e.g., engagement from the teachers’ perspective). We also recommend estimating objective academic demands (e.g., workload). Third, we focused on high school students who most probably experience the highest academic demands of all Chinese school students. The results cannot be generalized to other student groups (e.g., middle school students, vocational school students, and university students). The study design should therefore also be conducted with students from other academic backgrounds in the future. Fourth, the burnout theory origins in Western cultures. In the next step, cross-cultural comparisons between a Chinese sample and samples from Western countries should be conducted. As previously mentioned, grit may also have its dark side. Although perseverance of effort in goal pursuit is considered as a personal strength in the current study, we strongly encourage future research to take this aspect into consideration while investigating grit. To reveal the possible dark side, it may be useful to additionally specify the pursuing goal.

### Summary

The current study was the first attempt to transfer the JD-R model to Chinese school contexts with the integration of grit. The present study provides important implications for understanding students’ emotional responses to academic stressors. Our findings indicate that high academic demands may increase the risk of burnout and underlines that perseverance and long-term goal pursuance may prevent Chinese students from burnout and have the potential to contribute to their positive development.
