# Correction to: The Bright Side of Grit in Burnout-Prevention: Exploring Grit in the Context of Demands-Resources Model among Chinese High School Students

**DOI:** 10.1007/s10578-021-01198-3

**Published:** 2021-06-22

**Authors:** Ziwen Teuber, Fridtjof W. Nussbeck, Elke Wild

**Affiliations:** 1 Department of Psychology, Bielefeld University, Universitaetsstr. 25, 33615 Bielefeld, Germany; 2 Department of Psychology, University of Konstanz, Konstanz, Germany

## Correction to: Child Psychiatry & Human Development (2021) 52:464–476 10.1007/s10578-020-01031-3

The article “The Bright Side of Grit in Burnout-Prevention: Exploring Grit in the Context of Demands-Resources Model among Chinese High School Students”, written by Ziwen Teuber, Fridtjof W. Nussbeck, Elke Wild, was originally published electronically on the publisher’s internet portal (https://link.springer.com/article/10.1007/s10578-020-01031-3) on 28 July 2020 without open access. With the author(s)’ decision to opt for Open Choice, the copyright of the article changed on 23 May 2021 to © The Author(s) 2021 and the article is forthwith distributed under the terms of the Creative Commons Attribution 4.0 International License (http://creativecommons.org/licenses/by/4.0/), which permits use, duplication, adaptation, distribution and reproduction in any medium or format, as long as you give appropriate credit to the original author(s) and the source, provide a link to the Creative Commons license and indicate if changes were made.

The Open Access funding enabled and organized by Projekt DEAL.

The original article has been corrected.

